# Zika Virus—A Reemerging Neurotropic Arbovirus Associated with Adverse Pregnancy Outcomes and Neuropathogenesis

**DOI:** 10.3390/pathogens13020177

**Published:** 2024-02-15

**Authors:** Kenneth C. Elliott, Joseph J. Mattapallil

**Affiliations:** 1 Department of Microbiology & Immunology, The Henry M Jackson Foundation for Military Medicine, Uniformed Services University, Bethesda, MD 20814, USA; 2 Department of Microbiology & Immunology, Uniformed Services University, Bethesda, MD 20814, USA

**Keywords:** Zika, Dengue, pregnancy, neuropathology, CZS, ADE, cross-neutralization

## Abstract

Zika virus (ZIKV) is a reemerging flavivirus that is primarily spread through bites from infected mosquitos. It was first discovered in 1947 in sentinel monkeys in Uganda and has since been the cause of several outbreaks, primarily in tropical and subtropical areas. Unlike earlier outbreaks, the 2015–2016 epidemic in Brazil was characterized by the emergence of neurovirulent strains of ZIKV strains that could be sexually and perinatally transmitted, leading to the Congenital Zika Syndrome (CZS) in newborns, and Guillain-Barre Syndrome (GBS) along with encephalitis and meningitis in adults. The immune response elicited by ZIKV infection is highly effective and characterized by the induction of both ZIKV-specific neutralizing antibodies and robust effector CD8^+^ T cell responses. However, the structural similarities between ZIKV and Dengue virus (DENV) lead to the induction of cross-reactive immune responses that could potentially enhance subsequent DENV infection, which imposes a constraint on the development of a highly efficacious ZIKV vaccine. The isolation and characterization of antibodies capable of cross-neutralizing both ZIKV and DENV along with cross-reactive CD8^+^ T cell responses suggest that vaccine immunogens can be designed to overcome these constraints. Here we review the structural characteristics of ZIKV along with the evidence of neuropathogenesis associated with ZIKV infection and the complex nature of the immune response that is elicited by ZIKV infection.

## 1. Introduction

Zika virus (ZIKV) is a flavivirus that is primarily transmitted by *Aedes aegypti* mosquitoes and is endemic in the tropical regions of the world. It was first identified in 1947 from a sentinel rhesus macaque in the Zika forest by the Uganda Virus Research Institute in Entebbe, Uganda, Africa and characterized in 1952 [1,2]. The earliest cases of human infections were reported in 1954 in Nigeria. Serological evidence from India and South East Asia using ZIKV-specific neutralizing antibody tests suggests that infections likely spread to India and neighboring countries as early as 1954 [3]. The first major modern outbreak of ZIKV was recorded in 2007 in the Yap islands, Micronesia, with widespread infection and reports of the first cases of autochthonous transmission [4]. Between 2012 and 2014, seven confirmed cases of ZIKV were reported in Thailand [5], followed by a major outbreak in French Polynesia in 2013 [6], with subsequent transmission to the Americas, initially to Brazil and then to central America and the Caribbean islands [7]. Major outbreaks were reported in 2015 in Brazil that were associated with significant numbers of infants being born with microcephaly, indicating perinatal transmission from infected mothers to their fetus [8]. Subsequent studies provided clear evidence of sexual transmission of ZIKV [9]. By 2016, ZIKV had spread worldwide, with numerous countries recording confirmed cases of infections. The World Health Organization (WHO) declared a public health emergency of international concern in 2016 with the Centers for Disease Control issuing travel alerts to countries that reported major outbreaks of ZIKV. The rate of infections reported worldwide decreased by 2018–2019, potentially driven by the emergence of herd immunity [10,11,12].

Zika virus causes asymptomatic infection in most people, with about 25–50% of those infected experiencing a mild febrile illness characterized by acute viremia, fever, rash, conjunctivitis, and joint and muscle pain [4,13,14]. Infection is cleared rapidly, and symptoms abate in 1–2 weeks, though ZIKV RNA has been detectable in the urine and other body fluids for extended periods of time after initial exposure [15,16,17,18]. Infection during pregnancy, however, has been associated with higher incidence of Congenital Zika Syndrome (CZS), especially in women who are infected during the first trimester of pregnancy [19,20]. CZS is characterized by neuropathogenesis and microcephaly in fetuses, eye abnormalities, arthrogryposis, etc., and developmental defects in newborns [19,21,22]. Studies have shown that ZIKV was detectable in the amniotic fluid [23] and brains of stillborn fetuses [24] of mothers infected with ZIKV. Bell et al. [25] in 1971 demonstrated that ZIKV could readily replicate in neurons and astroglial cells of 1-day-old Webster Swiss mice following intracerebral infection with ZIKV. Rather surprisingly, there is little evidence of microcephaly in newborns with ancestral strains of ZIKV prior to 2015, suggesting that its ability to cross the transplacental barrier and cause neuropathology in fetuses was a more recently acquired trait. Additionally, ZIKV infection in some people has been associated with Guillain-Barre Syndrome (GBS), which is characterized by peripheral paralysis [26,27,28,29,30,31].

Though the rate and prevalence of ZIKV infections in recent years have not been reported at the scale seen during the 2015–2016 outbreak, the potential for reemergence of ZIKV and its threat to pregnant women remains high. Here we review the structural characteristics of ZIKV, the neurovirulence and immune responses induced by ZIKV, and the consequences of the cross-reactive immune responses induced by ZIKV against Dengue virus (DENV).

## 2. Zika Virus Structural and Non-Structural Proteins

**Figure 1:**
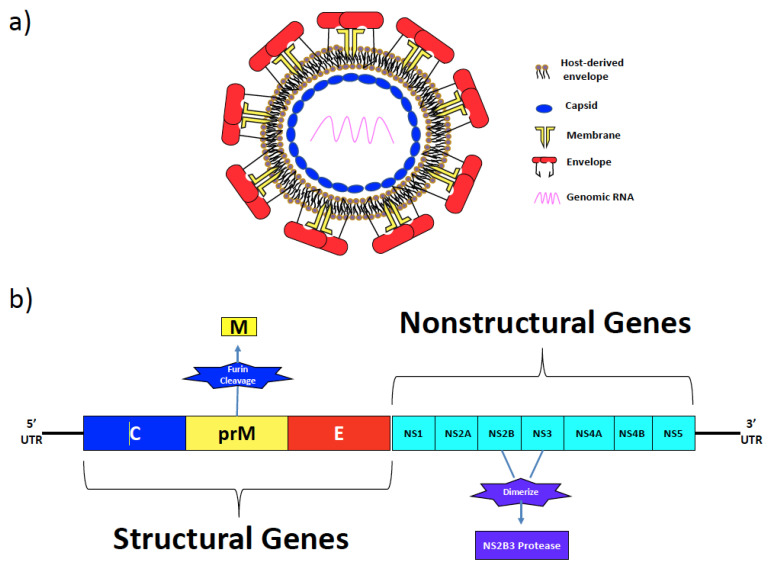
Cartoon structure of ZIKV and DENV particle and genome map. Zika virus and Dengue virus are members of the Flaviviridae family, containing a positive sense, single stranded ~10.5kb RNA genome. (**a**) ZIKV and DENV virions are enveloped and consist of three structural proteins, namely capsid (C), membrane (M), and envelope (E) proteins. Capsid proteins make up the icosahedral nucleocapsid that surrounds the genomic material. The M protein is only expressed on mature virions, it contains transmembrane regions, and is organized as a heterodimer underneath the E protein. The E protein contains the primary antigenic targets and is responsible for viral entry and assembly. (**b**) The genome encodes 3 structural genes (C, prM, E) and 7 nonstructural genes (NS1, NS2A, NS2B, NS3, NS4A, NS4B, NS5). NS2A assists with virion assembly through recruitment of the NS2BNS3 protease. NS2B dimerizes with NS3 to act as a protease and cleave the viral polypeptide.

Zika virus is an enveloped virus with a single-stranded, positive-sense RNA genome (~10.5 Kb) that encodes a 5′ and 3′ UTR, and a single open reading frame (ORF) [32] that codes for three structural proteins, namely the capsid (C), membrane (M), and the envelope (E) proteins, and seven non-structural (NS) proteins that include NS1, NS2A, NS2B, NS3, NS4A, NS4B, and NS5 (Figure 1a,b) [33]. The ORF is translated as a single polyprotein and cleaved by the cellular and viral proteases into the individual functional proteins during replication [34].

The lipid envelope of ZIKV is derived from the host endoplasmic reticulum during replication [35]. The E and M protein are embedded in the lipid envelope with the E protein on top of the M protein [36]. The E protein contains the primary antigenic targets for neutralizing antibodies and is responsible for receptor binding, viral assembly, and membrane fusion [37]. Expressed as a homodimer, the E protein has about 90 homodimers that are tightly arranged on the lipid envelope in a herringbone symmetry on the surface of mature virions, giving them a smooth surface [36]. Sitting below the E protein is the M protein (75 amino acids) that contains an N-terminal M loop along with a stem and two transmembrane regions that heterodimerize with the stem and transmembrane regions of the E protein [36]. The M protein is initially synthesized as a precursor (prM) protein that serves as a chaperone for the E protein during virion assembly. Once in the golgi, prM is either cleaved by furin protease to be expressed on mature virions as the M protein or as the uncleaved prM protein on immature virions [38,39,40]. Immature ZIKV has a rough spiky surface, unlike the smooth surface of mature virions, primarily due to the expression of prM on the viral envelope.

Within the heterodimeric E protein, each monomer (~505 amino acids) consists of an ectodomain in the N-terminus, a stem, and a transmembrane region in the C-terminus. The N-terminal ectodomain of the monomeric E protein has three subregions, namely EDI, EDII, and EDIII, that are displayed on the surface with the stem and transmembrane regions below it. The ectodomains play an important role in viral entry and are the primary targets for neutralizing antibodies. Of the three subdomains, EDI is arranged in the center and connects EDII to the EDIII domain. The EDI has a glycosylation site at N154 in the 150 loop that spans the amino acid residues 145–160, that was shown to be essential for ZIKV infectivity; the loss of glycan at N154 was associated with attenuation and reduced neuro invasiveness [41]. Other studies have suggested that the glycan loop in EDI likely plays a key role in the transmission of ZIKV [36]. The EDII domain mediates the dimerization of the E protein monomers and harbors the highly conserved fusion loop at the tip that contributes to fusion with the cell membrane during viral entry. Unlike EDI and EDII, the EDIII domain has an immunoglobulin-like structure, and contains the lateral ridge (LR) epitope along with the BC, DE, and FG loops. Numerous studies have reported that the EDIII domain harbors the receptor binding site, thereby making it a primary target for neutralizing antibodies [42,43,44,45,46,47]

The C protein is the main component of the icosahedral nucleocapsid that encapsulates the RNA genome and harbors 18 amino acids that serve as a signal peptide for the proper folding and expression of the E protein. Recent studies have implied that a single amino acid change at position 101 of the capsid (C) protein, from lysine to arginine (termed K101R), significantly enhanced the virulence of the African lineage of ZIKV [48].

Unlike other proteins encoded by the ZIKV genome, NS1 is a secreted glycoprotein with varying functions depending on its location [49,50]. Inside the cell, it acts as a cofactor for viral replication, whereas outside the cell it acts as an immune antagonist through degradation of complement proteins [51,52,53]. NS1 has been associated with vascular leakage and endothelial cell barrier disruption [54]. NS2A is responsible for virion assembly. It starts by recruiting the NS2B/NS3 protease and the C-prM-E, where the latter will be processed. After this processing, NS2A binds the viral genome, which allows for the assembly of a virion [49]. NS2B is a viral protease responsible for cleaving the viral polypeptide in addition to subverting the immune system through the inhibition of antiviral host factors. Interestingly, NS3 serves as the viral helicase during genome replication, in addition to having RNA triphosphatase activities. NS3 also dimerizes with NS2B, an important cofactor, to form the viral protease. NS4A is involved in cellular membrane modeling, inducing autophagy, enabling viral replication, and antagonizing the interferon response, while NS4B plays roles in viral replication and host immunomodulation [55]. NS5 is the viral RNA Polymerase that also has methyltransferase activity. The 5′ and 3′ UTRs are primarily involved in viral replication [56].

## 3. Epidemiology

Zika virus was first isolated in 1947 from a pyrexic sentinel monkey in the Zika forest during a surveillance for vector-borne viruses at the Uganda Virus Research Institute, located in Entebbe, Uganda, Africa, and was characterized in 1952. Since then, sporadic outbreaks of ZIKV were reported in Africa that spread eastward to Asia [3]. Smithburn et al. [3] reported that neutralizing antibodies to ZIKV were readily detectable in significant numbers of subjects in India in 1954, suggesting that ZIKV was likely prevalent in Asia during the 1950s. In 1953, cases of ZIKV were reported in Nigeria, though infection rates remained low with mild symptoms [57].

The first modern day large-scale outbreak of ZIKV was reported in 2007 on the island of Yap in the Federated States of Micronesia. Unlike the earlier outbreaks in Africa, the 2007 outbreak in the Yap islands was thought to be caused by an Asian isolate that was more pathogenic than previous outbreaks, suggesting a divergence from the African isolate [4,58]. Patients presented with rash, conjunctivitis, and arthralgia, but none of the current well-known neurological sequela such as CZS or GBS [4]. By 2012, ZIKV cases were reported in Thailand, followed by a major outbreak in the Pacific islands including French Polynesia, Easter Island, the Cook Islands, and New Caledonia in 2013-2014. The outbreak in French Polynesia lasted for 6 months with ~12% of the population reporting symptoms, and for the first time, a case of GBS was reported in the 7th week of the outbreak, suggesting that ZIKV had likely evolved to become neurotropic. During this outbreak, the first documented case of maternal fetal transmission of ZIKV was reported, along with the detection of ZIKV in semen, though these cases were rare [6]. Unlike the previous outbreaks, the 2012–2016 outbreak was predominantly caused by the Asian lineage [5,6,7]. In May of 2015, initial infections were recorded in Brazil, with lineages that were phylogenetically linked to the new epidemic strain of Asian lineage introduced in 2013. The outbreak continued to amplify in Brazil, and by 2016, it is estimated that between 200,000 and 1.3 million cases were reported in Brazil, with seroprevalence rates of over 63% [8,59,60]. During the peak of the epidemic, in the United States (US), all 49 states along with the District of Columbia reported confirmed cases of ZIKV infection, though most of these cases (4944) were travel-related, with only 224 cases being reported as autochthonous infections [61,62,63,64,65].

Unlike the US mainland, the US territories reported over 36,000 locally transmitted cases and about 145 imported cases. Among the US territories, Puerto Rico reported as many as 39,717 confirmed cases between 1 November 2015 and 31 December 2016 [66], though the true scale was hard to assess due to the lack of resources needed for constant surveillance. This was further complicated by the asymptomatic nature of infection, which made it difficult to track new infections without rigorous screening. The circulation of other flaviviruses also added a layer of complexity to the diagnosis as they caused similar non-specific symptoms. The ZIKV epidemic waned by 2017–2018. Since then, sporadic outbreaks have been reported in Asia and the Americas, though most of these cases remained largely localized. In 2022, the Pan American Health Organization (PAHO) reported that Brazil recorded 3,238 confirmed cases of ZIKV, suggesting that the potential for outbreaks remains high in endemic areas (https://www3.paho.org/data/index.php/en/mnu-topics/zika-weekly-en/ accessed on 11 January 2024).

## 4. Neuropathogenesis and Clinical Outcomes

Zika virus infection in most people has been associated with mild symptoms [31] such as maculopapular rash, low-grade fever, general malaise, headache, muscle and joint aches, and arthritis-like symptoms [4]. A meta-analysis of 73 case studies found that the most common symptoms included exanthema (89%), arthralgia (63%), fever (62%), conjunctivitis (45%), myalgia (48%), headache (46%), and diarrhea (13%), with most infected people recovering from infection in 5–7 days. Fatal cases and hospitalizations were rare, 0.1% and 11%, respectively, but a majority of these cases were associated with comorbidities or GBS [67].

However, unlike past infections worldwide, the 2015–2016 outbreak in Brazil recorded the emergence of neurovirulent strains of ZIKV that could replicate in the placenta and cross the transplacental barrier to infect the fetus, leading to a spectrum of birth defects [13,21,68,69,70,71,72,73]. In contrast to other congenital infections, CZS has been associated with a number of fetal abnormalities that were found to be specific to congenital ZIKV infection such as severe microcephaly, ocular damage characterized by macular scarring and retinal mottling, arthrogryposis, multifocal calcifications in the cortex and subcortical white matter, associated cortical displacement, and mild focal inflammation, and early hypertonia. These conditions lead to functional deficits such as dysphagia and adverse clinical outcomes such as epilepsy during the early stages of life [19,22,24]. During the 2015–2016 outbreak, Brazil reported over 8000 cases of microcephaly and other neurological disorders associated with congenital ZIKV infection. A number of other countries experiencing ZIKV transmission reported a similar association between ZIKV and CZS at the same time [74,75]. The risk for developing CZS was reported to be significantly higher when pregnant women were infected during the 1st trimester (11% of cases) as compared to the 3rd trimester of pregnancy [76,77]. Congenital ZIKV infection has also been associated with stillbirth and miscarriages along with neonatal death in a subset of pregnant women. Paixao et al. [78] reported that infants born with CZS to ZIKV-infected mothers had an 11-times greater risk of death during the first three years after birth with higher risk correlating with infection during the 1st trimester of pregnancy [76] and the severity of microcephaly [79]. Costa et al. reported a case fatality rate of 10% in early life for infants born with CZS [80].

Most ZIKV infections in adults are asymptomatic, though serious “neurologic sequelae” have been reported in ~0.3% of cases, with ~75% of them being GBS [67], which is characterized by damage to peripheral nerves thought to be caused by an aberrant autoimmune response triggered by ZIKV infection, leading to muscle and bilateral limb weakness and in some cases paralysis [81]. Approximately 20–30% of GBS patients are likely to suffer from respiratory failure that requires ventilation assistance. While symptoms may peak within 4 weeks, recovery takes months or years with supportive care [82]. Increased incidence of GBS has been reported in areas with high ZIKV transmission rates [26,29,83]. Dirlikov et al. reported that the GBS Passive Surveillance System (GBPSS) instituted by the Puerto Rico Department of Health and CDC during the outbreak of 2016 identified 61% of suspected patients with GBS that were likely infected with ZIKV [29]. Likewise, during the 2013–2014 outbreak of ZIKV in French Polynesia, all subjects with GBS had ZIKV-specific neutralizing antibodies [31]. Additionally, a higher incidence of GBS was reported in areas where ZIKV prevalence was high [27,28,30].

ZIKV infection has been associated with other neurological sequela such as myelitis and meningoencephalitis. Mecharles et al. [84] reported on the case of a 15-year-old girl who presented to the emergency clinic in Guadeloupe with pain in the left arm, frontal headaches, and conjunctival hyperaemia 7 days prior to admission. On day 9 post-admission, her spinal MRI showed lesions in the cervical and thoracic regions of the spinal cord. High concentrations of ZIKV were detected in the cerebrospinal fluid (CSF) along with serum and urine, suggesting neurotropism. Carteaux et al. [85] reported a similar case in an 81-year-old man who had been admitted to the ICU within 10 days after being on a 4-week cruise. He was found to be febrile and comatose with hemiplegia of the left side and paresis of the right upper limb, and MRI examination of the brain showed signs of meningoencephalitis. Analysis of CSF on day 1 after admission was positive for ZIKV by PCR and culture, suggesting ZIKV as the primary cause for meningoencephalitis in this patient. Other studies have reported similar cases of CNS infection and the presence of ZIKV in the CSF of these patients [86,87].

The exact reasons why ZIKV became neurotropic is still under investigation. Studies comparing the ZIKV genomes from the 2015 outbreak to earlier isolated strains have identified mutations within specific regions of the genome that may have contributed to the increased teratogenicity of ZIKV. Yuan et al. [88] reported that a single amino acid mutation in the prM protein of ZIKV from serine to asparagine (S139N) was associated with an increase in neurovirulence and mortality in newborn mice as compared to the ancestral Asian strains. Likewise, Liu et al. [89] identified a mutation in the NS1 protein of ZIKV in the 2016 strain (A188V) that increased transmission to mosquitos as compared to the 2010 strain. Others have shown that a single mutation in the E protein of ZIKV (V473M) in the 2013 strains increased neurovirulence and viral loads in the placenta and fetal brains of mice as compared to the 2010 strain [90]. Whether these single amino acid mutations were sufficient to drive the neurovirulence of the contemporary strains in humans is still under investigation. Wongsurawat et al. [91] observed that the ZIKV strain isolated from a microcephalic patient lacked the S139N mutation in prM reported by Yuan et al. [88]. Other studies have reported increased pathogenicity and disease severity in animal models infected with African strains [92,93,94,95], suggesting that a combination of mechanisms likely contributed to the enhanced teratogenicity observed during the 2015–2016 outbreak of ZIKV.

## 5. Zika Virus Infection and Immune Responses

### 5.1. Type I Interferon Response

**Figure 2:**
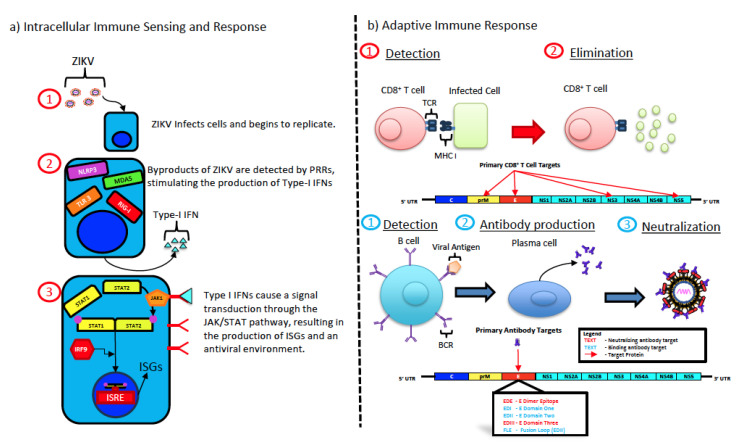
Innate and adaptive immune response to ZIKV infection. (**a**) ZIKV infects target cells through receptor-mediated endocytosis and replicates in the cytoplasm. Replication intermediates such as single stranded and double stranded RNA are sensed by pathogen recognition receptors such as NLRP3, MDA5, TLR3, and RIG-I, which drives the activation and phosphorylation of IRF3 and IRF7, which translocate to the nucleus and drive the production of Type I IFNs. These Type I IFNs will be exported from the cell and interact with receptors on neighboring cells, leading to the phosphorylation and activation of the JAK/STAT pathway. Activation and phosphorylation of the JAK/STAT pathway leads to the recruitment of IRF9, which will translocate to the nucleus and bind to the Interferon Stimulated Response Element (ISRE), leading to the transcription of over 100 interferon stimulated genes (ISG) that induce an antiviral state. (**b**) Adaptive immune responses are characterized by the activation of ZIKV-specific CD8 cytotoxic T cells that recognize epitopes in the context of MHC Class I and eliminate infected cells through the release of the perforin and Granzyme B. ZIKV-specific CD8 T cell responses have been shown to recognize epitopes located within the prM, E, NS3, and NS5 proteins. ZIKV-specific adaptive B cell responses have been mapped to a number of proteins with neutralizing antibody responses primarily targeting the EDIII domain along with the E dimer epitope.

Immune responses play a critical role in controlling ZIKV infection (Figure 2a,b). In healthy adults, infection is self-limiting and initial viremia is resolved within 5–10 days after infection. Type I interferon responses have been shown to play a major role in protection from ZIKV infection, as demonstrated in mice that lack either IFNα or the receptors for IFNα [96,97,98,99]. Bulstrode et al. demonstrated that IFNβ produced by myeloid cells significantly restricts ZIKV infection of progenitor cells in primary tissue explants [100]. Though Type I IFNs clearly suppress ZIKV infection, they have also been shown to play an ambivalent role. Da Silva et al. reported that increased expression of IFNα and IFNβ during the early stages of ZIKV infection in humans was correlated with higher viremia [101]. Others have demonstrated a significant increase in the secretion of pro-inflammatory Type I IFN-stimulated cytokines and chemokines, namely CXCL8, CXCL10, CCL2, and IL-1RA, that correlated with worse symptoms in some patients [102]. Likewise, Kam et al. observed a significant association between high levels of Type I IFN-stimulated cytokines such as CCL2 and CXCL10 following ZIKV infection and fetuses with congenital abnormalities [103]. Hayashida et al. [104], using both immunocompetent and deficient mouse models, noted that infection of the CNS with ZIKV induced Type I IFN responses that failed to prevent the establishment of infection and encephalitis.

Induction of Type I IFN during ZIKV infection is mediated through intracellular sensing by pattern recognition receptors (PRR). Numerous PRRs such as the Toll-like receptor-3 (TLR3), retinoic acid-inducible gene 1 (RIG-I), NLRP3, and melanoma differentiation-associated gene 5 (MDA5) have been shown to detect ssRNA or dsRNA byproducts of ZIKV replication to initiate the induction of Type I IFN responses [105,106]. Interestingly however, numerous reports have documented the interactions between ZIKV proteins and innate sensors and/or downstream signaling that interfere with the induction of innate IFN responses during ZIKV infection.

TLR3 is an endosomal PRR that recognizes single stranded RNA and its activation has been shown to drive innate responses to ZIKV infection, though whether it plays a protective role is less clear. Plociennikowska et al. reported that TLR3 activation suppressed IFN responses induced through the RIG-I pathway despite eliciting a proinflammatory cytokine response during ZIKV infection [106]. Others have shown that TLR3-mediated immune responses induced by ZIKV infection were associated with the depletion of neural progenitor cells in an organoid model [107]. Likewise, Ojha et al. reported that TLR3 activation was accompanied by an increase in ZIKV replication and inflammation in primary human astrocytes [108].

Numerous studies have examined the interaction between ZIKV and various innate sensors. RIG-I is activated by ZIKV dsRNA, which then interacts with the mitochondrial antiviral signaling protein (MAVS), leading to an IFN response that is capable of suppressing ZIKV replication [109]. On the other hand, Hu et al. reported that ZIKV NS4A could antagonize the RIG-I-MAVS signaling pathway by interacting with the CARD-TM domains, leading to an attenuated interferon response [110]. Likewise, MDA5 activation by ZIKV also signals through MAVS, suggesting a synergistic role with RIG-I in the induction of innate IFN responses. Knockdown of MDA5 was found to significantly increase ZIKV replication and viral titers in Sertoli cells, indicating a role for MDA5 in protection from ZIKV infection [109]. Interestingly, Reidel et al. demonstrated that the interaction of ZIKV NS3 with scaffolding proteins 14-3-3ϵ and 14-3-3η prevented the translocation of RIG-I and MDA5 from the cytosol to the mitochondria, thereby preventing activation of the MAVS pathway [111]. Interaction of the NLRP3 inflammasome with ZIKV NS5 during replication has been shown to activate Caspase-1, leading to the production of IL-1beta, which plays a role in driving the acute inflammation during ZIKV infection [112,113], though others have reported that ZIKV NS3 downregulates the NLRP3 pathway in macrophages [114].

The activation of PRRs by ZIKV leads to the phosphorylation of IRF3 and IRF7, key transcription factors that drive the production of type I IFN during infection [115,116]. Lazear et al. reported that infection of mice that lacked IRF3/5/7 with ZIKV was associated with significant loss of body weight and mortality as compared to control mice [116]. Subsequent studies have, however, shown that ZIKV NS2A inhibits full-length, regulatory domain-deficient, and constitutively active IRF3, whereas NS4A inhibited full-length or regulatory domain-deficient IRF3 [117,118].

Binding of secreted IFN to its receptors IFNAR1 and IFNAR2 on cells leads to the activation of the JAK/STAT pathway and the induction of several ISGs capable of suppressing ZIKV replication [119]. However, Wang et al. reported that upregulation of ISG15 during ZIKV infection was associated with enhanced viral replication [120]. Others have shown that ZIKV antagonizes the JAK/STAT pathway in humans through the accelerated degradation of STAT2 in NS5-independent and dependent mechanisms [121,122]. Recent studies suggest that STAT1 is also a target for degradation during ZIKV infection [123].

Altogether, the above studies suggest that ZIKV has evolved mechanisms that interfere with the activation of numerous PRRs and downstream signaling events, leading to a muted or suppressed Type I IFN response [124] that in turn supports ZIKV replication.

### 5.2. Cell-Mediated Immune Responses

Natural killer (NK) cells are a key component of the innate cell-mediated immune response to viral infections. NK cells recognize and eliminate virally infected cells in a variety of ways such as the absence of an inhibitory signal, the antibody being bound to infected cells, or cellular stress. The role of NK cells during ZIKV infection is still under investigation. Studies have reported an increase in the frequencies of NK cells during the acute stages of infection that correlated with a decrease in the post-peak decline of ZIKV viremia, suggesting a role for NK cells in the early containment of infection [125]. Other studies have demonstrated that NK cells residing in the placenta (decidual NK cells) recognize ER stress, downregulate NK cell-inhibitory receptors on ZIKV-infected trophoblasts in the placenta, and kill these infected cells, thereby preventing ZIKV transmission across the placenta [126]. On the other hand, Dudley et al. reported that persistent viremia was established in pregnant rhesus macaques despite the robust activation of NK cells [127], though this may be related to the immunosuppressive microenvironment during pregnancy. Moucourant et al. [128] showed that NK cells from a cohort of female subjects who were acutely infected with ZIKV harbored activated and terminally differentiated NK cell subsets that produced significant levels of IFNγ and TNFα without marked cytotoxic responses. On the other hand, Lum et al. [129] noted that infection of cells isolated from peripheral blood of healthy subjects was associated with secretion of IFNγ and an increase in CD107a, the marker for degranulation. There is little evidence to show that ZIKV interferes with NK cell activity in vivo, though Glasner et al. reported that ZIKV infection upregulated MHC I expression on infected cells in vitro, allowing these cells to escape detection by NK cells [130]. During pregnancy, regulatory NK cells predominate within the placenta and it is not clear what role these NK cells play in either the protection or pathogenesis of ZIKV infection in women who get infected during pregnancy. Additional studies are needed to address this question.

Unlike NK cells, adaptive CD8^+^ T cell responses play a central role in antigen-specific recognition and clearance of virally infected cells. Numerous studies have demonstrated that CD8^+^ T cell responses were indispensable for protection during ZIKV infection. ZIKV infection induces a strong CD8^+^ effector response and ~20% of ZIKV antigen-experienced CD8^+^ T cells were found to express IFNγ and TNFα when stimulated with PMA and ionomycin, and to express higher levels of T-bet [131,132]. Others have shown high levels of antigen-specific CD8^+^ T cells containing Granzyme B in WT C57BL/6 mice following infection with ZIKV [133]. Hassert et al. reported that the depletion of CD8^+^ T cells in ZIKV-infected mice was associated with an increase in lethality when compared to control mice, showing that CD8^+^ T cells were essential for protection [134]. Likewise, HLA-B*0702 *ifnar1^−/−^* mice immunized with an NS3 vaccine that was designed to generate a CD8^+^ T cell response rather than a neutralizing antibody response were protected from death or fetal growth restriction, with decreased viral loads in the serum, brain, and liver tissue three days post-infection. Additionally, CD8 knockout mice were found to be highly susceptible to infection, suggesting that the ability to mount a CD8^+^ T cell response is essential for the control of ZIKV [135,136]. Ngono et al. reported that prM, E, and NS5 proteins harbored the primary protective, immunodominant epitopes involved in the CD8^+^ T cell response in mice [136].

CD8^+^ T cell responses have also been shown to play a critical role in protecting against sexual transmission of ZIKV. Scott et al. reported that CD4^+^-depleted or B cell-deficient mice immunized subcutaneously with ZIKV and subsequently challenged intervaginally had reduced viral loads in the vagina and uterine horns compared to control mice, showing that vaccine-induced adaptive CD8^+^ responses contributed to protection [137]. Blocking of Type I IFN responses in CD4^+^-depleted or B cell-deficient mice with anti-IFNAR antibodies did not alter the protective effect of adaptive CD8 T cell responses that were induced following vaccination. Additionally, neutralizing antibodies were undetectable in the vaginal washes of these mice at the time of infection, supporting the predominant role of CD8^+^ T cells in protection from infection [137]. Other studies have reported a role for CD8^+^ T cells in protection from CNS pathology during ZIKV infection. Adoptive transfer of CD8^+^ T cells from ZIKV-immunized wildtype mice into naive *Ifnar^−/−^* mice was associated with a significant decrease in viral burden in the brain and spinal cord following ZIKV infection that correlated with an increase in the number of CD8^+^ cells in the brain and spinal cord [138]. Likewise, Nazerai et al. demonstrated that immunocompetent mice that were initially infected intravenously with ZIKV and reinfected intracranially 4 weeks later were protected from CNS infection; viral control was lost when CD8^+^ T cells were depleted, demonstrating a protective role for CD8^+^ T cells [133]. Data from nonhuman primate models have largely supported the findings in mouse models. Dudley et al. showed that CD8^+^ T cell expansion during acute stages of ZIKV infection correlated with a decrease in serum viral loads in rhesus macaques [127], whereas depletion of CD8^+^ T cells was associated with higher levels of ZIKV RNA in lymph nodes and the spleen [139].

CD4^+^ T cells play a critical role in the induction of immune responses by providing help to B and T cells. Depletion of CD4^+^ T cells was accompanied by a decrease in the magnitude of plasma cells and germinal center (GC) B cell responses, and reduced ZIKV E protein-specific neutralizing IgG levels at 7 days post-infection, suggesting that ZIKV-specific B cell responses were compromised in the absence of CD4 T cells [140]. Likewise, Hassert et al. reported that the depletion of CD4^+^ cells was associated with enhanced lethality and neuropathology, along with increased viral loads in the brain, spinal cord, and liver of ZIKV-infected *Ifnar^−/−^* mice [141]. Interestingly, Lucas et al. showed that CD4^+^ T cells interact with B-cells in an IFNγ-dependent manner, leading to the production of neutralizing IgG2a antibodies that protected mice from lethal infection with ZIKV, whereas the depletion of CD4^+^ T cells significantly impaired these responses [142], suggesting that critical CD4^+^ T cell helper responses were essential for the generation of ZIKV-specific antibody-mediated control of infection.

### 5.3. Humoral Immune Response

Adaptive B cell responses are essential for protection during ZIKV infection. In human patients, both ZIKV-specific IgM and IgA responses were found to peak between 7 and 14 days after infection, after which they rapidly declined, whereas IgG responses peaked around day 21 and remained high for up to 2 years [143,144]. Most ZIKV-specific neutralizing antibodies appear to target the EDIII domain and the E dimer epitope (EDE), with EDI or EDII having modest-to-no neutralizing ability. Stettler et al. showed that an EDIII monoclonal antibody with an altered Fc region, ZKA64-LALA, could protect A129 mice from lethal ZIKV infection when given one day before or after infection [44]. Zhao et al. showed that two separate monoclonal antibodies that target the lateral ridge (LR) epitope in the EDIII domain were protective in mice, and decreased viral loads and prevented weight loss and death in the case of lethal infection [45]. Magnani et al. used an NHP model to show that a cocktail of monoclonal antibodies derived from a human patient was able to prevent infection when given prophylactically one day prior to ZIKV infection. Over the course of 21 days, ZIKV was not detected in any of the monkeys receiving the treatment. The lack of a specific IgM and IgG response post-infection further suggested that the antibody cocktail provided sterilizing immunity [145]. Sankhala et al. isolated a number of neutralizing monoclonal antibodies from an RM infected with ZIKV that could be broadly separated into four antigenic groups targeting the cross-promoter epitopes within the E protein [146]. Interestingly, the only epitope that all the antibodies came into competition for was the quaternary antigen E dimer epitope (EDE), suggesting that this target is extremely immunogenic [146]. Interestingly, a study by Wang et al. reported that the use of an engineered bispecific antibody (FIT-1) targeting EDII and EDIII was more effective at preventing viral escape than the EDII-only targeted monoclonal antibody when tested in vitro. Additionally, FIT-1 was found to be protective when passively transferred to A129 mice, increasing survivability and decreasing viral load in a time and dose-dependent manner [147].

Neutralizing antibody responses are vital for protection of the fetus and the prevention of placental insufficiency. In marmosets, vaccination was shown to prevent vertical transmission of ZIKV through a neutralizing antibody response which lasted for up to 18 months [148]. Sapparapu et al. reported that a potent human antibody, ZIKV-117, given prophylactically or therapeutically to pregnant mice reduced viral loads in both maternal and fetal tissues. As expected, the decrease in viral burden resulted in decreased damage to the placenta, decreased trophoblast cell death, and increased fetal body weight [43].

## 6. Cross-Reactivity with Dengue

**Figure 3:**
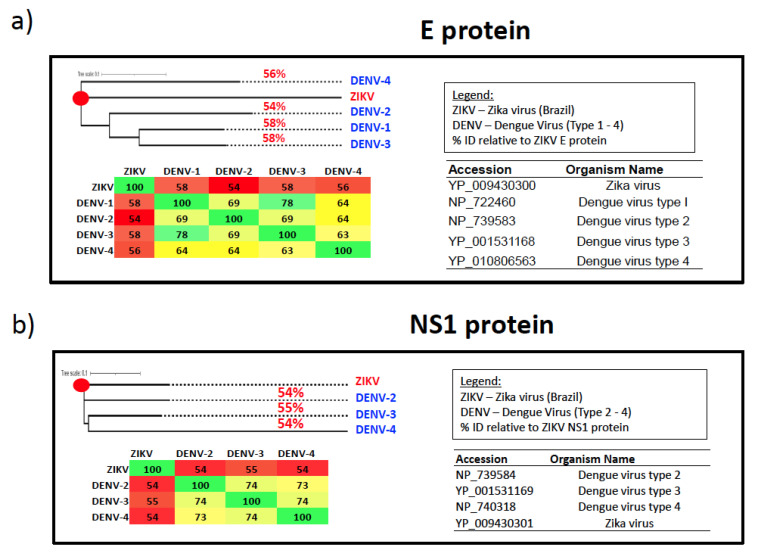
Sequence similarity between ZIKV and DENV E and NS1 proteins. (**a**) Phylogenetic tree and heat map showing the relatedness of ZIKV and DENV-1—4 E protein based on amino acid alignment. The NCBI Virus database [159] (https://www.ncbi.nlm.nih.gov/labs/virus/vssi/#/ accessed on 30 October 2023) was used to download the reference sequences for E proteins of ZIKV and DENV-1—4. The sequences were then aligned using the online Clustal Omega Multiple Sequence Alignment (MSA) Tool [160] (https://www.ebi.ac.uk/Tools/msa/clustalo/ accessed on 30 October 2023). The resulting output was then pasted into the online “Simple Phylogeny” tool [160] https://www.ebi.ac.uk/Tools/phylogeny/simple_phylogeny/ accessed on 30 October 2023). The phylogenic tree was then uploaded to iTOL [161] (https://itol.embl.de/ accessed on 30 October 2023) for visual enhancement and scaling. A heat map for amino acid identity was created from the Clustal Omega MSA. (**b**) Phylogenetic tree and heat map showing the relatedness of ZIKV and DENV-1–4 NS1 protein based on amino acid alignment. The NCBI Virus database [159] (https://www.ncbi.nlm.nih.gov/labs/virus/vssi/#/ accessed on 30 October 2023) was used to download the reference sequences for the NS1 proteins of ZIKV and DENV-2–4; no reference sequence was available for DENV-1 NS1. As such, the exact similarity between ZIKV NS1 and DENV1 is not available. The sequences were then aligned using the online Clustal Omega Multiple Sequence Alignment Tool [160] (https://www.ebi.ac.uk/Tools/msa/clustalo/ accessed on 30 October 2023). The resulting output was then pasted into the online “Simple Phylogeny” tool [160] (https://www.ebi.ac.uk/Tools/phylogeny/simple_phylogeny/ accessed on 30 October 2023). The phylogenetic tree was then uploaded to iTOL [161] (https://itol.embl.de/ accessed on 30 October 2023) for visual enhancement and scaling. The heat map for amino acid identity was created from the Clustal Omega MSA using Excel.

Recent studies have shown that antigenic cross-reactivity between ZIKV and related flaviviruses such as DENV (Figure 3a,b) could lead to the immune-mediated enhancement of disease. George et al. [149] in 2017 was the first to demonstrate that prior immunity to ZIKV significantly enhanced DENV-2 infection in rhesus macaques. The enhancement of DENV infection correlated with the induction of significant levels of DENV cross-reactive binding antibodies by ZIKV that failed to cross-neutralize DENV. A number of studies in animal models and humans that followed were found to confirm these initial findings [150,151,152,153]. Fowler et al. [154] in 2018, using mouse models, reported that maternally acquired ZIKV antibodies enhanced the severity of DENV disease. Katzelnick et al. [155] in 2020 reported that ZIKV infection significantly enhanced the future risk of severe DENV disease in human pediatric cohorts in Nicaragua. Rather surprisingly, pre-existing immunity to DENV was not found to enhance ZIKV infection and disease [156]. A recent study by Kim et al. [157], however, suggests that like the ZIKV-mediated enhancement of DENV, pre-existing DENV immunity could potentially enhance infection of ZIKV; DENV-immune pregnant marmosets were found to have significantly high ZIKV viral loads in the placenta and fetal tissues that correlated with the induction of ZIKV cross-reactive binding antibodies by DENV infection that failed to cross-neutralize ZIKV. Zambrana et al. [158] reported that primary exposure to ZIKV significantly increased the risk for symptomatic DENV infection with DENV 2–4 serotypes.

Though the potential of ADE cannot be minimized, several studies have reported the induction of cross-neutralizing antibodies. Barba-Spaeth et al. reported that a subset of mAbs that targeted a conformational epitope located within the EDE region of the DENV E protein had significant neutralizing activity against ZIKV [162]. On the other hand, a cross-reactive antibody that targeted the FLE region neutralized DENV with minimal-to-no neutralizing activity against ZIKV [162]. Fernandez et al. demonstrated that DENV E-dimer epitope-specific human mAbs had therapeutic efficacy against ZIKV infection, protected mice from lethal infection, and reduced viral loads in the serum, brain, testis, epididymis, and eye [163]. Wang et al. reported that 13 circulating human serum antibodies from a ZIKV patient in Venezuela reacted with the ZIKV soluble envelope protein with varying strengths. However, only two were found to be non-cross-reactive with DENV, while also having highly neutralizing activity against ZIKV. Interestingly, a highly neutralizing antibody targeting ZIKV, named Z20, was capable of neutralizing DENV-1 to 4 when present in high concentrations [164].

These studies suggest that both DENV and ZIKV harbor epitopes capable of cross-neutralizing each other that could be harnessed using structure-based design strategies to develop immunogens that can prevent both ZIKV and DENV infections and minimize the potential for ADE, as several preclinical studies have reported [165,166,167,168,169,170]. There are currently 13 ZIKV-specific vaccine candidates that are in either Phase I or II clinical trials [171]. Additionally, vaccines that can protect against multiple arboviruses have been explored using either chimeric virus vaccines [172,173] or mosquito saliva proteins as potential immunogens [174,175,176,177,178,179] to elicit pan-arboviral protection, although immunization with some salivary proteins has been associated with increased pathogenesis [180]. The preclinical and clinical success of mRNA vaccines against ZIKV and other arboviruses [166,181,182,183,184,185] shows that this vaccine platform could be used to develop multivalent flavivirus-specific vaccines, as has been shown for influenza [183,184] and SARS-CoV-2 [185].

In summary, the evolution of ZIKV from a benign pathogen to a more neurovirulent strain has raised significant public health concerns worldwide given its potential to cross the placenta and cause severe fetal neuropathology and CZS. As herd immunity wanes, the potential for future ZIKV outbreaks remains high and there is urgency to develop vaccination strategies that can protect pregnant women and their unborn fetus from infection and CZS. Though cross-reactivity with other flaviviruses poses an immediate constraint on the development of a safe and efficacious vaccine, the identification of cross-neutralizing antibodies raises hope that these constraints can be overcome using novel structure-based vaccine design strategies.
